# Effects of Acute Maximum-Intensity Exercise on Matrix Metalloproteinase-2, -9, and Tissue Inhibitor of Metalloproteinase-1 Levels in Adult Males with Type 1 Diabetes Mellitus Treated with Insulin Pumps

**DOI:** 10.3390/jcm13237077

**Published:** 2024-11-23

**Authors:** Joanna Kryst, Bartłomiej Matejko, Olga Czerwińska-Ledwig, Łukasz Tota, Roxana Zuziak, Anna Piotrowska

**Affiliations:** 1 Department of Chemistry and Biochemistry, Institute for Basics Sciences, Faculty of Physiotherapy, University of Physical Education in Kraków, 31-571 Kraków, Poland; olga.czerwinska@awf.krakow.pl (O.C.-L.); roxana.zuziak@awf.krakow.pl (R.Z.); anna.piotrowska@awf.krakow.pl (A.P.); 2 Department of Metabolic Diseases, Jagiellonian University Medical College, 30-688 Kraków, Poland; b.matejko@uj.edu.pl; 3 Metabolic Diseases and Diabetology Clinical Department, University Hospital in Krakow, 30-688 Kraków, Poland; 4 Department of Physiology and Biochemistry, Faculty of Physical Education and Sport, University of Physical Education in Kraków, 31-571 Kraków, Poland; lukasz.tota@awf.krakow.pl

**Keywords:** diabetes mellitus, T1DM, physical activity, metalloproteinases

## Abstract

**Background:** Dysregulation of matrix metalloproteinases (MMPs) activity is considered one of the potential causes of vascular complications in diabetic patients. Since training volume may influence MMPs levels in varying ways, the aim of our study was to evaluate changes in MMPs levels following acute maximum-intensity exercise in male patients with type 1 diabetes mellitus (T1DM). **Methods:** This study included 24 male T1DM patients and 10 healthy controls. Aerobic capacity was evaluated with a treadmill test. Levels of matrix metalloproteinase-2 (MMP-2), matrix metalloproteinase-9 (MMP-9), and tissue inhibitor of metalloproteinase-1 (TIMP-1) were measured both before the aerobic capacity test and 60 min after its completion utilizing enzyme-linked immunosorbent assay (ELISA) system kits. **Results:** Before the aerobic capacity test only, MMP-9 serum levels were significantly elevated in the T1DM group compared to the controls. Following maximum-intensity exercise, the levels of MMP-2, MMP-9, and TIMP-1 were significantly higher in T1DM patients than in the control group. Between-group comparisons revealed that maximum-intensity exercise induced a statistically significant increase in MMP-2 serum levels from baseline in T1DM patients compared to controls. **Conclusions:** Our findings suggest that high-intensity exercise in T1DM patients leads to dysregulation of MMPs, as manifested by a significant increase in MMP-2 levels. This dysregulation may play a role in the development of vascular complications in diabetic patients.

## 1. Introduction

Type 1 diabetes mellitus (T1DM) is associated with an increased risk of cardiovascular disease (CVD) [1,2], which is the leading cause of mortality in the diabetic population [3]. Among the pathophysiological mechanisms underlying CVD development in T1DM, impaired regulation of extracellular matrix (ECM) turnover by matrix metalloproteinases (MMPs) has been postulated [4]. The increased activity of MMPs observed in CVD has been associated with pathological degradation and remodeling of extracellular matrix proteins, contributing to atherosclerosis, plaque rupture [5], and diabetic nephropathy, a common microvascular complication of diabetes [4]. MMPs belong to a family of zinc-dependent endopeptidases involved in the degradation of various protein components of the ECM and its remodeling. Endogenous tissue inhibitors of metalloproteinases (TIMPs) regulate MMPs activity, and an imbalance between MMPs and TIMPs is observed in various pathological conditions [6]. Among the 23 MMPs expressed in human tissues, two (MMP-2 and MMP-9) are classified as gelatinases [7]. Both are involved in the pathophysiology of diabetes complications, primarily cardiovascular complications [8]. 

In T1DM patients, elevated levels of MMPs and TIMP-1 have been reported, and these are associated with hyperglycemia, low-grade inflammation, and endothelial dysfunction [9]. Dysregulation of gelatinase activity in T1DM patients has also been previously documented [10,11,12]. Moreover, studies in both adult [13] and pediatric patients with T1DM [10] have reported that elevated plasma levels of MMP-2 are associated with a higher risk of cardiovascular complications. The association between plasma levels of TIMP-1 and MMP-9 and the symptoms of vascular complications in T1DM has also been suggested [14].

Physical activity plays a crucial role in metabolic control in T1DM. In this patient population, regular physical activity has been shown to improve not only blood glucose and lipid metabolism [15] but, most importantly, to reduce the risk of severe diabetic retinopathy [16], progression of kidney disease, cardiovascular risk, and premature mortality [17]. For adult patients with TIDM, the American Diabetes Association (ADA) recommends at least 150 min of moderate-to-vigorous intensity activity spread evenly throughout the week [18]. However, T1DM is not a contradiction to even more intense physical activities, such as high-endurance sports [19]. Physical activity in T1DM may increase the risk of an uncontrolled drop in blood glucose levels, with aerobic exercise often inducing hypoglycemia in diabetic patients taking exogenous insulin [20]. On the other hand, high-intensity exercise can lead to hyperglycemia accompanied by hypoinsulinemia and ketonuria, particularly in untrained patients [21]. Thus, the metabolic and neuroendocrine responses in T1DM patients can vary depending on the type of physical exercise performed [22].

In healthy individuals, mismatched training volume can modulate the MMPs levels inconsistently with the intended purpose. In an animal model of resistance training, the activity of MMP-2 and MMP-9 in plasma, skeletal muscle, and visceral adipose was differently regulated by different training volumes [23]. In humans, studies on muscle biopsies have shown that acute exercise stimulates MMP-9 gene transcription, while repeated training is necessary to increase mRNA levels of MMP-2 and TIMP-1/2. While acute exercise induces an elevation in serum or plasma levels of MMP-9 [24], results from repeated training have been more inconsistent, with some studies observing no change or a trend toward a reduction in blood levels of both gelatinases [24,25]. 

It is important to note that the studies mentioned did not include patients with T1DM. To the best of our knowledge, the regulation of MMPs levels in T1DM patients induced by physical activity has not yet been described in the literature. 

Understanding exercise-induced responses in the body is crucial for establishing appropriate physical activity recommendations for T1DM patients. Our aim was to assess the effects of acute maximum-intensity exercise on serum levels of MMP-2, MMP-9, and TIMP-1 in T1DM male patients treated with a personal insulin pump, compared to healthy controls. We hypothesize that the proposed high-intensity exercise may increase gelatinase levels, which may already be dysregulated by T1DM itself.

## 2. Materials and Methods

### 2.1. Study Participants 

This study included 24 male patients with T1DM treated with a personal insulin pumps and 10 healthy male controls matched for age and BMI. The inclusion criteria for diabetes patients were adults with T1DM treated with a personal insulin pump, no significant cardiovascular, respiratory, or locomotor system disorder, and the most recent HbA1c level ≤ 9%. Exclusion criteria for T1DM participants included chronic complications of diabetes (pre-proliferative or proliferative retinopathy, autonomic neuropathy, and polyneuropathy, stage 3 or higher chronic kidney disease); severe hypoglycemia or diabetic ketoacidosis within the week before the test; significant cardiovascular, respiratory or locomotor system disorders; glycemia before a standardized meal > 250 mg/dL or <60 mg/dL or <70 mg/dL when accompanying hypoglycemic symptoms occurred; postprandial glycemia after 90 min > 300 mg/dL and presence of ketone bodies in urine; body mass index (BMI) ≥ 35 kg/m^2^; abnormalities in the resting electrocardiography; lack of qualification by an internal medicine physician. 

The patients were informed about the details of the study protocol and provided written consent to participate. Participants were allowed to withdraw from the study at any time without consequence. This study followed the principles of the Declaration of Helsinki and was approved by the Jagiellonian University Bioethics Committee (approval No. 1072.6120.113.2017 of 28 September 2017). The exercise tests were performed under the constant supervision of a physician.

### 2.2. Data Collection

Fasting measurements of anthropometric parameters were performed on the day of the exercise testing, and all the measurements were conducted by the same researcher. Body mass (BM), body height (BH), body fat mass (FM), fat-free mass (FFM), total body water (TBW), and body cell mass (BCM) were established. Body mass was measured with a body composition analyzer (Tanita BIA-547, TANITA, Tokyo, Japan; accuracy: 0.1 kg and 0.1%), body height was assessed with a SECA-210 stadiometer, and body composition was determined with the bioelectric impedance analysis using the AKERN BIA 101 analyzer (Akern, Pisa, Italy). 

The aerobic capacity level test was carried out on a mechanical treadmill (Saturn 250/100R, h/p/cosmos, Nussdorf-Traunstein, Germany). During the 4 min warm-up at the beginning of the test, the participant marched at a speed of 7 km h^−1^ with an inclination angle of 1°. The running speed was then increased by a 1.0 km h^−1^ increment every 2 min. The test was stopped when the participant refused to continue due to utmost fatigue or reached a point at which an increase in running speed resulted in no increase in monitored parameters (heart rate and VO_2_). The test was performed under the supervision of a physician.

A detailed description of the exercise test, along with data regarding nutrition assessment and glycemia control during the test, has been published previously [26,27].

Blood samples were collected before the aerobic capacity test and 60 min after its completion. Blood samples were collected in clot-activator-containing tubes. The samples were then centrifuged at 2000 rpm, and the serum was separated from the clot. The obtained serum was stored frozen at −80 °C until analysis (no longer than 2 months). Moreover, 24 h before the biochemical analysis, samples were stored at −20 °C and then transferred to 4 °C one hour prior to analysis. Each serum sample was used only once to avoid the effect of repeated freezing on studied proteins. Commercially available enzyme-linked immunosorbent assay (ELISA) system kits (SunRedBio, Shanghai, China) were used in accordance with the manufacturer’s recommendations to determine the serum MMP-2 (ref no: 201-12-0905, assay range: 12–3000 ng/mL), MMP-9 (ref no: 201–12-0937, assay range: 2–600 ng/mL), and TIMP-1 (ref no: 201-12-1237, assay range: 2–600 ng/mL) concentrations.

### 2.3. Statistical Analysis 

All statistical analyses were performed using GraphPad Prism, version 9.4.1 (GraphPad Software Inc., San Diego, CA, USA). The normality of the data was assessed using the D’Agostino and Pearson tests. Based on the results, either the student’s *t*-test or the Mann–Whitney test was used to compare data between TD1M patients and the control group. A paired *t*-test was used to compare data within the same group of patients before and after the test. Spearman correlation analysis was conducted to assess correlations between variables in the experimental group (T1DM patients). Differences were considered statistically significant for a *p*-value < 0.05.

## 3. Results

Table 1 shows the anthropometric characteristics of the experimental group (T1DM patients) and the control group. No significant differences were found between groups regarding anthropometric measures; however, a difference was revealed in the patient’s age.

The experimental group was characterized by suboptimal metabolic control of 7.1 ± 1%, with a mean diabetes duration of 11.8 ± 6.2 years and an average duration of treatment with a personal insulin pump of 6.9 ± 4.2 years (Table 2).

As shown in Table 3, at baseline, only MMP-9 serum levels were significantly elevated in the T1DM group compared to controls. Baseline MMP-2 and TIMP-1 levels did not differ significantly between the two groups. However, after maximum-intensity exercise, the levels of all three analyzed biomarkers were significantly higher in T1DM subjects than in the control group. 

After maximum-intensity exercise, serum levels of MMP-2 significantly increased from baseline in T1DM patients but not in control subjects. No significant changes in MMP-9 or TIPM-1 values were observed after maximum-intensity exercise compared to baseline values in either T1DM or control subjects. Between-group comparisons revealed that maximum-intensity exercise induced a statistically significant increase in MMP-2 serum levels from baseline in T1DM patients compared to controls, while exercise-induced changes in MMP-9 and TIMP-1 levels did not differ significantly between T1DM and controls (Table 3). 

In T1DM patients, no correlation was found between maximum-intensity exercise-induced changes in the analyzed biomarkers. Regarding the correlation of changes in MMPs and TIMP-1 values with anthropometric parameters, positive correlations were observed between exercise-induced changes in MMP-9 and TIMP-1 serum levels with body mass, body cell mass, and fat-free mass, as well as between changes in TIMP-1 serum levels and fat mass. No correlation was found between exercise-induced changes in MMP-2 values and any of the analyzed anthropometric indices. Among clinical and physiological indications, there was a negative correlation between changes in MMP-9 levels and maximal oxygen consumption (VO_2_ max) (Table 4).

## 4. Discussion

Our results confirm previously reported differences in the baseline concentration of MMP-9 in T1DM patients compared to healthy individuals. For the first time, we report specific changes in the concentration of selected matrix metalloproteinases following maximum-intensity exercise in T1DM subjects. The results suggest that maximum-intensity exercise has an unfavorable effect on the concentration of the studied metalloproteinases, potentially increasing the rate of tissue degeneration. 

Consistent with our results, higher serum or plasma levels of MMP-9 [12,28,29,30,31] have been reported in T1DM patients compared to healthy subjects. In the literature, significantly higher levels of MMP-9 in diabetic vs. healthy individuals have also been detected in salivary samples [32]. 

Higher levels of MMP-2 have also been reported in several studies [10,11,12,31]; however, in our study, the difference did not reach statistical significance. Other studies in this field have shown conflicting results, with some authors reporting no differences in MMP-9 [10,33] or MMP-2 levels [28,29] between T1DM patients and controls. When discussing this inconsistency, it should be noted that, with the exception of the Thrailkill 2007/2009 studies [11,33], the research reported no statistical significance in gelatinase levels between T1DM patients and control subjects recruited relatively small sample sizes (less than 50 patients). Moreover, pre-analytical methods in sample preparation could introduce bias in the measurement of gelatinase levels. Since MMP-9 is released from blood cells, and both leukocytes and platelets contain high concentrations of MMP-9, the duration of the interval between blood sampling and plasma/serum separation from the blood cell mass can influence the obtained results [34]. Alternatively, the observed inconsistency could stem from differences in the populations included in the studies. For example, the study by Derosa et al. (2005) was conducted in a pediatric population over a 5-year follow-up, during which MMP-9 levels increased with age in both T1DM patients and controls [10]. This suggests that tissue remodeling associated with growth may influence gelatinase levels independently of diabetes. 

As our patients were free of clinical vascular complications, the results suggest that MMP-9 levels are elevated in T1DM patients before the significant onset of complications. Impaired glycemic control in diabetes can directly influence MMPs levels. It has been shown that glucose intake increases MMP-2 and MMP-9 plasma concentrations [35], while insulin decreases MMP-9 levels in non-diabetic individuals [36]. Furthermore, a recent study has shown enhanced expression of MMP-2 in response to high glucose stimulation in a diabetic mouse heart model [37]. Dysregulation of metalloproteinases in diabetic patients may also result from comorbidities. T1DM is associated with autoimmune diseases [38], and both upregulation and downregulation of MMP-9 have been reported in a variety of diseases with autoimmune origin [39].

Elevated baseline levels of MMP-9 and MMP-2 are suggested to be associated with vascular complications in diabetes [13,14]. In our population of relatively young T1DM patients, baseline MMP-9 levels were significantly higher compared to controls, which raises the possibility that pathological remodeling of the ECM may have already occurred in the T1DM patients included in our study. Therefore, this group of patients should avoid physical activity that leads to further elevation of the analyzed MMPs. However, after maximal-intensity exercise, the levels of all three analyzed biomarkers (MMP-2, MMP-9, and TIMP-1) were significantly increased in T1DM patients compared to controls. The treadmill test performed until exhaustion particularly affected MMP-2 levels in T1DM patients, an effect that was not observed in healthy control subjects. 

One possible explanation for the obtained results could be the higher oxidative and nitrosative stress indicators reported in our study population of T1DM patients compared to controls [26]. The pathogenesis of diabetes mellitus and its complications may be related to oxidative stress. Moreover, reactive oxygen species have been suggested to increase MMP-9 activity [40]. In the context of patient training, it is important to note that transient oxidative stress is induced by high-intensity exercise [41,42] and that exercise intensity influences oxidative responses [43]. Additionally, in an animal model of T1DM, low-intensity exercise training rather than high-intensity exercise reduced the elevated levels of MMP-2 in skeletal muscles [44]. It could be speculated that in a patient population with an overproduction of reactive oxygen and nitrogen species due to chronic hyperglycemia, its further increase as a result of high-intensity exercise may lead to an increase in gelatinase levels in diabetic patients. In the present study, no changes in MMP-2 levels were reported in the control group following the proposed exercise training. Similarly, according to the literature, which is mainly based on healthy subjects, no changes in MMP-2 levels following an acute exercise have been reported in most studies [24]. Our results suggest that the observed increase in MMP-2 levels after the proposed training may be specific to T1DM subjects. Thus, we hypothesize that in the population of young adult T1DM patients, diabetes-induced dysregulation of gelatinases leads to its further elevation following an acute high-intensity exercise. Of note, T1DM patients participating in this study showed significantly lower aerobic capacity compared to controls, despite satisfactory glycemic control [26], which could also exacerbate the response to high-intensity exercise. 

A study was recently published where large-scale proteomic profiling was performed in healthy subjects after acute exercise. The authors found no significant changes in MMP-2 protein 1 h post-exercise compared to baseline. However, one hour after exercise ended, dysregulation of proteins involved in glucose homeostasis was detected [45]. Thus, it could be suggested that dysregulation in glucose metabolism in diabetic patients may also modify their biochemical response to exercise. 

As a result of intense physical exercise, muscle damage can occur, including cell death, damage to sarcomeres, and the associated infiltration of inflammatory cells. During maximal exercise, Mi et al. (2023) showed a significant increase in dystroglycan (DAG_1_) [45], which is a component of transmembrane linkage between the extracellular matrix and the cytoskeleton in skeletal muscle [46]. Upregulation of DAG_1_ observed at peak exercise may be due to contraction-induced injury, initiating the remodeling of the extracellular matrix, in which metalloproteinases are involved. Gelatinases, which are involved in the hydrolysis of proteins forming the basal lamina surrounding the myofiber sarcolemma, initiate tissue reorganization processes aimed at repairing damage [24]. 

Since the measurements of MMPs levels were performed only once, 60 min after the test, we can only speculate whether the values returned to baseline during recovery from maximal-intensity exercise. Both MMP-2 and MMP-9 are implicated in vascular damage processes and are associated with kidney damage [47], making their elevation particularly unfavorable in diabetic patients. Elevated baseline gelatinase levels in T1DM patients put this group of patients at an increased risk for vascular and kidney complications. Additionally, increased MMP-2 has been suggested to be associated with diabetic retinopathy [48] through pathological retinal neovascularization [49].

Although the between-group comparison showed a statistically significant difference in MMP-9 levels before and after maximal-intensity exercise, no significant increase in MMP-9 levels after exercise was observed when the groups were analyzed separately. This is surprising, as most studies in healthy subjects have reported increased MMP-9 levels following an acute exercise [24]. One possible explanation is that the 60 min point after the test may have been too short to capture significant changes in MMP-9 levels. Mackey et al. (2004) demonstrated a slow increase in serum concentrations of MMP-9, with statistical significance only being reached 8 days after the acute exercise [50]. In the study by Suhr et al. (2007), although a significant elevation in MMP-9 was reported 60 min after acute exercise, a further significant increase was observed 3 h later [51]. Alternatively, the low detection level of MMP-9 in our study, compared to other research reporting serum MMP-9 levels in T1DM and healthy subjects [29,30], should be mentioned. 

## 5. Study Limitations

Among the limitations of our study, the relatively small sample size should be noted. Gelatinase levels were measured in serum, while some authors suggest that citrate plasma is more appropriate for measuring circulating MMP-2 and MMP-9 [34]. Additionally, measuring MMPs activity would provide a more comprehensive understanding of its regulation in T1DM patients compared to controls. The lack of assessment of MMPs levels a few hours after the capacity test was another limitation of our research. With sufficient funding, future studies should include later time points of assessments to verify whether the elevated MMP-2 levels return to baseline during recovery from maximal-intensity exercise.

## 6. Conclusions

Intensive exercise can lead to muscle damage and a stress response, which depends on an individual’s physical condition and any existing comorbidities. In T1DM patients with reported lower aerobic capacity and impaired glucose homeostasis, the biochemical response to high-intensity exercise may differ from that of the healthy population. This difference can, in turn, impact disease progression and the development of complications. Therefore, guidelines for physical activity in T1DM patients, particularly for high-intense exercise, should consider molecular responses to optimize safety for this population.

## Figures and Tables

**Table 1:** Anthropometric characteristics of the study population with statistical analysis between groups (Student’s *t*-test or the Mann–Whitney test).

	T1DM (N = 24)	Control (N = 10)	*p*
Age [years]	23.1 (5.4)	23.4 (1.9)	0.0376
BH [cm]	179.8 (8.7)	181.2 (6.1)	0.6534
BM [kg]	78.7 (15.0)	72.5 (8.7)	0.2350
BCM [kg]	37.3 (5.3)	36.1 (3.7)	0.375
FFM [kg]	64.5 (8.5)	61.2 (6.2)	0.2792
FM [kg]	14.2 (8.2)	11.4 (3.5)	>0.9999
TBW [%]	60.7 (4.8)	57.0 (14.2)	0.4316

Data are presented as means (standard deviation); T1DM—type 1 diabetes mellitus; BH—body height; BM—body mass; BCM—body cell mass; FFM—fat-free mass; FM—fat mass; TBW—total body water.

**Table 2:** Clinical and physiological characteristics of T1DM patients.

	T1DM (N = 24)
DM duration [years]	11.8 (6.2)
Time of CSII use [years]	6.9 (4.2)
HbA1c [%]	7.1 (0.9)
DDI [IU]	57.6 (16.6)
VO_2_ max *	44.7 (6.0)
CGM	156.1 (30.6)

Data are presented as means (standard deviation); T1DM—type 1 diabetes mellitus; DM—diabetes mellitus; CSII—continuous subcutaneous insulin infusion (treatment with a personal insulin pump); HbA1c—glycated hemoglobin; DDI—daily dose of insulin; CGM—continuous glucose monitoring; * VO_2_ max (mL kg^−1^ min^−1^)—maximal oxygen consumption.

**Table 3:** Levels of MMP-2, MMP-9, and TIMP-1 before and after maximum-intensity exercise in T1DM and control subjects with statistical analysis (a paired *t*-test for before-and-after comparison within the same group; Mann–Whitney test for comparison between groups).

	T1DM		*p*	Control		*p*	*p* T1DM vs. Control	
	Before	After		Before	After		Before	After
MMP-2 [ng/mL]	229.3 (136.8)	267.0 (174.0)	<0.0001	158.8 (101.6)	154.8 (100.0)	0.8457	0.0949	0.01
Δ	37.7 (47.5)			−4.0 (24.8)			0.0017	
MMP-9 [ng/mL]	58.6 (32.5)	60.4 (34.9)	0.4732	37.7 (21.1)	35.1 (17.6)	0.1602	0.0146	0.0046
Δ	1.8 (7.9)			−2.6 (4.9)			0.1179	
TIMP-1 [ng/mL]	42.2 (28.3)	44.4 (28.4)	0.6401	24.5 (19.4)	23.2 (18.9)	0.2637	0.0606	0.0287
Δ	2.2 (22.5)			−1.3 (4.4)			0.1074	

Data are presented as means (standard deviation); T1DM—type 1 diabetes mellitus; MMP-2—matrix metalloproteinase 2; MMP-9—matrix metalloproteinase 9; TIPM-1—tissue inhibitor of matrix metalloproteinase-1.

**Table 4:** Spearman correlation results.

Indices	*p*	r
Biochemical indices		
ΔMMP-2 vs. Δ MMP-9	0.1894	0.2774
ΔMMP-2 vs. Δ TIMP-1	0.1263	0.3209
ΔMMP-9 vs. Δ TIMP-1	0.2296	0.2548
Anthropometric indices		
ΔMMP-2 vs. BM	0.4023	0.1791
ΔMMP-2 vs. BCM	0.2976	0.2218
ΔMMP-2 vs. FM	0.4307	0.1687
ΔMMP-2 vs. FFM	0.6892	0.0861
ΔMMP-9 vs. BM	0.0127	0.5009
ΔMMP-9 vs. BCM	0.0013	0.6171
ΔMMP-9 vs. FM	0.0663	0.3809
ΔMMP-9 vs. FFM	0.0066	0.5391
ΔTIMP-1 vs. BM	0.0055	0.5487
ΔTIMP-1 vs. BCM	0.0060	0.5436
ΔTIMP-1 vs. FM	0.0093	0.5191
ΔTIMP-1 vs. FFM	0.0146	0.4922
Clinical and physiological indices		
ΔMMP-2 vs. disease duration	0.0831	−0.3691
ΔMMP-2 vs. treatment duration (CSII)	0.3998	−0.1843
ΔMMP-2 vs. HbA1c	0.4441	0.1639
ΔMMP-2 vs. VO_2_ max *	0.8957	−0.0283
ΔMMP-2 vs. CGM	0.9775	−0.0061
ΔMMP-9 vs. disease duration	0.1568	−0.3052
ΔMMP-9 vs. treatment duration (CSII)	0.9964	−0.00099
ΔMMP-9 vs. HbA1c	0.8885	−0.0302
ΔMMP-9 vs. VO_2_ max *	0.0453	−0.4123
ΔMMP-9 vs. CGM	0.9743	0.0070
ΔTIMP-1 vs. disease duration	0.1013	−0.3503
ΔTIMP-1 vs. treatment duration (CSII)	0.8504	0.0416
ΔTIMP-1 vs. HbA1c	0.7448	0.0701
ΔTIMP-1 vs. VO_2_ max *	0.2229	−0.2583
ΔTIMP-1 vs. CGM	0.8275	0.0470

MMP-2—matrix metalloproteinase 2; MMP-9—matrix metalloproteinase 9; TIPM-1—tissue inhibitor of matrix metalloproteinase-1; BM—body mass; BCM—body cell mass; FM—fat mass; FFM—fat-free mass; CSII—continuous subcutaneous insulin infusion (treatment with a personal insulin pump); HbA1c—glycated hemoglobin; CGM—continuous glucose monitoring; * VO_2_ max (mL kg^−1^ min^−1^)—maximal oxygen consumption.

## Data Availability

The datasets generated and analyzed during the current study are available from the corresponding author upon reasonable request.
